# Characterization of a β-Adrenergic-Like Octopamine Receptor in the Oriental Fruit Fly, *Bactrocera dorsalis* (Hendel)

**DOI:** 10.3390/ijms17101577

**Published:** 2016-09-22

**Authors:** Hui-Min Li, Hong-Bo Jiang, Shun-Hua Gui, Xiao-Qiang Liu, Hong Liu, Xue-Ping Lu, Guy Smagghe, Jin-Jun Wang

**Affiliations:** 1 Key Laboratory of Entomology and Pest Control Engineering, College of Plant Protection, Southwest University, Chongqing 400715, China; huiminli0815@yahoo.com (H.-M.L.); jhb8342@swu.edu.cn (H.-B.J.); Shunhuagui@163.com (S.-H.G.); liukf@foxmail.com (X.-Q.L.); liuhong741@yeah.net (H.L.); luxueping91@163.com (X.-P.L.); guy.smagghe@ugent.be (G.S.); 2 Department of Crop Protection, Ghent University, Ghent 9000, Belgium

**Keywords:** biogenic amine, *Bactrocera dorsalis*, octopamine receptor, cyclic AMP, agonist, antagonist, functional expression, stress

## Abstract

The biogenic amine octopamine plays a critical role in the regulation of many physiological processes in insects. Octopamine transmits its action through a set of specific G-protein coupled receptors (GPCRs), namely octopamine receptors. Here, we report on a β-adrenergic-like octopamine receptor gene (*BdOctβR1*) from the oriental fruit fly, *Bactrocera dorsalis* (Hendel), a destructive agricultural pest that occurs in North America and the Asia-Pacific region. As indicated by RT-qPCR, *BdOctβR1* was highly expressed in the central nervous system (CNS) and Malpighian tubules (MT) in the adult flies, suggesting it may undertake important roles in neural signaling in the CNS as well as physiological functions in the MT of this fly. Furthermore, its ligand specificities were tested in a heterologous expression system where *BdOctβR1* was expressed in HEK-293 cells. Based on cyclic AMP response assays, we found that BdOctβR1 could be activated by octopamine in a concentration-dependent manner, confirming that this receptor was functional, while tyramine and dopamine had much less potency than octopamine. Naphazoline possessed the highest agonistic activity among the tested agonists. In antagonistic assays, mianserin had the strongest activity and was followed by phentolamine and chlorpromazine. Furthermore, when the flies were kept under starvation, there was a corresponding increase in the transcript level of *BdOctβR1*, while high or low temperature stress could not induce significant expression changes. The above results suggest that *BdOctβR1* may be involved in the regulation of feeding processes in *Bactrocera dorsalis* and may provide new potential insecticide leads targeting octopamine receptors.

## 1. Introduction

Octopamine, a biogenic amine, affects many diverse processes in invertebrate physiology, and has significant similarities between the octopaminergic signaling system in invertebrates and the adrenergic system of vertebrates [1]. It plays a key role in the insect nervous system as a neuromodulator, neurotransmitter, and neurohormone. For example, octopamine modulates behaviors such as aggression, sleep, oviposition, food-seeking, and locomotion [2].

Octopamine exerts its effects by binding to specific receptors that belong to the superfamily of G-protein coupled receptors (GPCRs), which share a common structural hallmark of seven transmembrane domains (TMs) and are associated with extracellular (ECLs) and intracellular loops (ICLs). The classification system of different octopamine receptors is based on the similarities of these proteins to vertebrate adrenergic receptors in terms of amino acid sequence and signaling pathway. According to the classification, octopamine receptors are grouped into three classes: α-adrenergic-like octopamine receptors (OctαRs), β-adrenergic-like octopamine receptors (OctβRs), and octopamine/tyramine (or tyraminergic) receptors (TyrRs) [3]. The activation of OctαRs primarily leads to an increase in intracellular Ca^2+^ concentration ([Ca^2+^]_i_) and a small increase in the level of intracellular cAMP ([cAMP]_i_), whereas the activation of OctβRs causes a moderate increase in [cAMP]_i_ but no increase in [Ca^2+^]_i_ [3,4], and the activation of TyrRs results in a reduction of [cAMP]_i_ and the generation of Ca^2+^ signals. Structural and pharmacological similarities of the TyrRs have been shown with vertebrate α_2_-adrenergic receptors. The TyrRs was preferent to be activated by tyramine to induce a decrease in [cAMP]_i_ [3,5].

Identifying the properties of octopamine receptors could contribute to the understanding of the specific functions of the octopaminergic system in insects. Since the isolation of the first octopamine receptor in the fruitfly *Drosophila melanogaster*, many additional receptors have been characterized in insects. In *D. melanogaster*, three subtypes of OctβRs have been identified. Among them, β_3_-octopamine receptor (OctβR3) plays a role in metamorphosis by regulating ecdysone synthesis [6], and OctβR2 has a strong effect on oviposition [7,8]. Two octopamine receptor subtypes have been isolated in *Bombyx mori* [9] and a partial sequence of a putative OctβR in the desert locust *Schistocerca gregaria* (*SgOctβR*) has also been cloned [10]. In addition, a β-adrenergic-like octopamine receptor from the rice stem borer (*Chilo suppressalis*) was characterized in a work that also provided evidence that CsOctβR2 is a likely mediator of locomotion [2]. Moreover, it has also been suggested that the octopaminergic system is associated with resource defense, alternative mating tactics, social tolerance, and indirect parental care in *Nicrophorus* *vespilloides* [11].

The oriental fruit fly, *Bactrocera dorsalis* (Hendel), is one of the most economically significant and widespread pests in the world, causing damage to over 250 different types of fruits and vegetables [12]. *B. dorsalis* has greatly expanded its geographical distribution adaptability also due to a powerful reproductive ability [13]. *B. dorsalis* has evolved high levels of resistance against most commonly used insecticides [14]. Therefore, it is urgent to discover novel targets for the development of insecticides. Fortunately, recent findings have shown that octopamine receptors possess potential for novel insecticide development. In the present study, we cloned a β-adrenergic-like octopamine receptor (*BdOctβR1*) from *B. dorsalis*. Quantitative Reverse Transcription-PCR (RT-qPCR) was used to investigate its expression profile over different developmental stages and tissues. Subsequently, with the use of a heterologous expression system, the pharmacological properties of *BdOctβR1* were determined for agonistic and antagonistic activity with typical ligands. Additionally, we report on the association between *BdOctβR1* and unfavorable (high and low) temperatures or starvation. We believe our works on the characterization of octopamine receptors in *B. dorsalis* may offer insights into the development of novel leads to control pest insects.

## 2. Results

### 2.1. Cloning and Sequence Analysis of β-Adrenergic-Like Octopamine Receptor (BdOctβR1)

**Figure 1:**
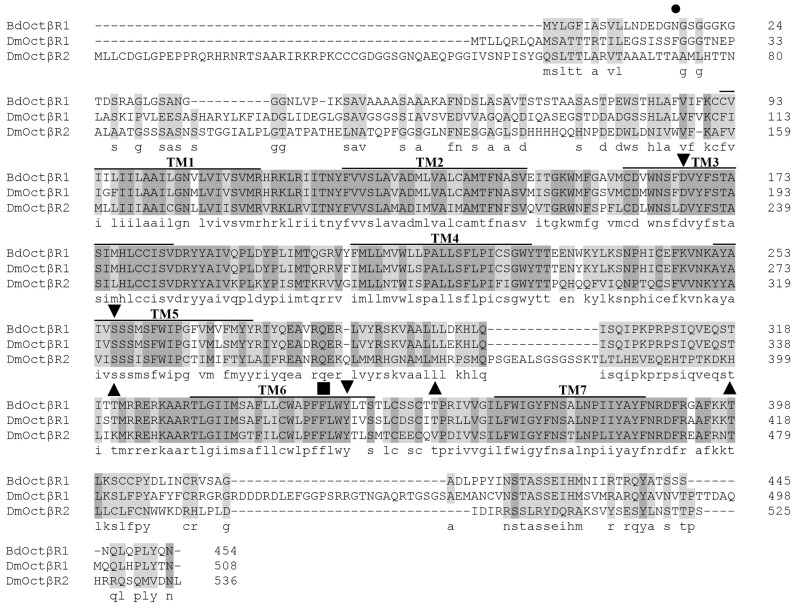
Amino acid sequence alignment of *BdOctβR1* and two β-adrenergic-like receptors from *Drosophila melanogaster* (*DmOctβR1* and *DmOctβR2*). TM, transmembrane domains. The seven transmembrane domains are numbered as TM1–7. The shaded sequences highlight the identity level of amino acids between the receptors. Identical amino acids are highlighted in dark gray and conserved amino acids are on a light gray, as determined using the 50% majority rules. Dashes are gaps that were introduced for alignment. Potential phosphorylation sites for protein kinase C are labeled with ▲ and Potential *N*-glycosylation sites indicated by are ●. Amino acids which are labeled with ▼ are predicted to be involved in octopamine binding. The second phenylalanine after the FxxxWxP motif in TM6, which is a unique feature of aminergic receptors, is indicated by ■.

The full length cDNA sequence of *BdOctβR1* was obtained by RT-PCR. The open reading frame (ORF) consists of 1365 bp encoding a 454 amino acid protein (GenBank accession number: XP_011212557). The putative amino acid sequence of *BdOctβR1* contains seven transmembrane domains, which is a signature of GPCRs. Multiple sequence alignment with *DmOctβR1* and *DmOctβR2* showed that *BdOctβR1* has highly conserved cysteine residues in the ECLs II and III, which form a disulfide bridge that is important in stabilizing the functional receptor structure. *BdOctβR1* also has the highly conserved DRY sequence at the cytoplasmic end of transmembrane domain III, which is considered important in G-protein coupling. In addition, it contains the NP(L/I)IY motif located within TM7 that is required for ligand-induced internalization and is conserved in all adrenergic receptors. Compared with the other two receptors, *BdOctβR1* has a relatively shorter N-terminal region than that in *D. melanogaster* (Figure 1).

**Figure 2:**
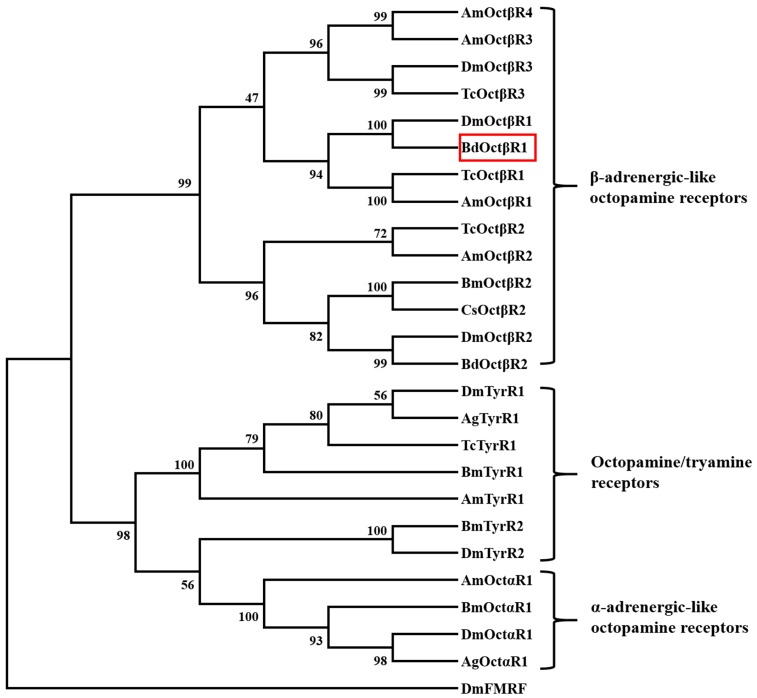
Phylogenetic tree of BdOctβR1 (red frame) and various biogenic amine receptors. Neighbor-joining tree was constructed in MEGA 5 using 1000 bootstrap tests re-sampling. The numbers at the nodes of the branches represent the level of bootstrap support for each branch. Ag: *Anopheles gambiae*; Am: *Apis mellifera*; Bd: *Bactrocera dorsalis*; Bm: *Bombyx mori*; Cs: *Chilo suppressalis*; Dm: *Drosophila melanogaster*; Tc: *Tribolium castaneum*. The receptor sequences followed by their GenBank accession numbers are listed in the order illustrated: *AgOctαR1*, EAA06361; *AgTyrR1*, EAA07468; *AmOctαR1*, NP_001011565; *AmOctβR1*, *XP_397139*; *AmOctβR2*, *XP_396348*; *AmOctβR3*, *XP_003249152*; *AmOctβR4*, *CCO13925*; *AmTyrR1*, NP_001011594; *BdOctβR1*, XP_011212557; *BdOctβR2*, JAC42311; *BmOctαR1*, BAF33393; *BmOctβR2*, BAJ06526; *BmTyrR1*, BAD11157; *BmTyrR2*, BAI52937; *CsOctβR2*, AEO89318; *DmFMRF*, NP_647758; *DmOctαR1*, NP_732541; *DmOctβR1*, Q9VCZ3; *DmOctβR2*, Q4LBB9; *DmOctβR3*, Q4LBB6; *DmTyrR1*, BAB71788; *DmTyrR2*, NP_650652; *TcOctβR1*, NP_001280514; *TcOctβR2*, NP_001280501; *TcOctβR3*, XP_008198078; *TcTyrR1*, NP_001164312. The FMRFamide receptor of *D. melanogaster* was used as an outgroup.

Furthermore, phylogenetic analysis was conducted with the octopamine receptors from other insect species. The phylogenetic tree clustered into three groups, consisting of α-adrenergic-like octopamine receptors, β-adrenergic-like octopamine receptors, and octopamine/tyramine receptors (Figure 2). *BdOctβR1* grouped with β-adrenergic-like octopamine receptors, which have been shown to be functional receptors for octopamine. *BdOctβR1* showed a very close relationship to *DmOctβR1* of *D. melanogaster* and *TcOctβR1* of *Tribolium castaneum*.

### 2.2. Developmental Stages and Tissue-Specific Expression Pattern

**Figure 3:**
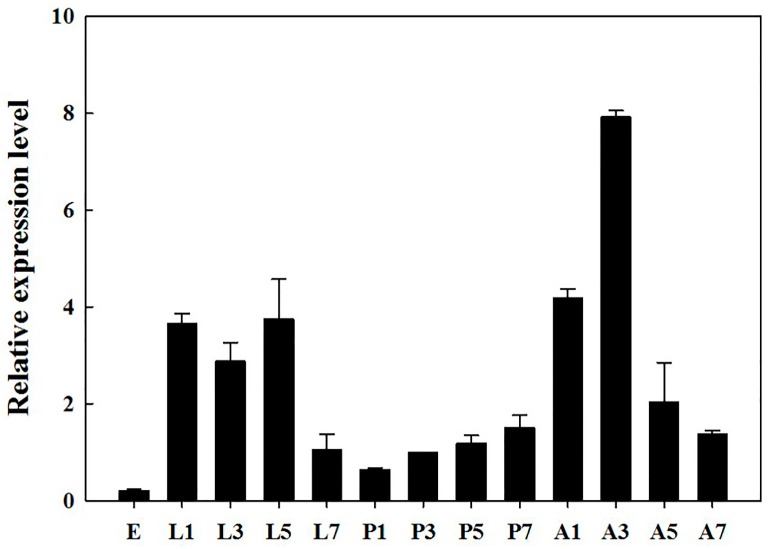
Relative expression levels of *BdOctβR1* at different developmental stages. Different stages are displayed by E (egg), L (larva), P (pupa), and A (adult). Different numbers 1, 3, 5, 7 represent days 1, 3, 5, and 7 of the developmental stage, respectively. The data shown are mean ± standard error (S.E.) (*n* = 3), normalized relative to *α-tubulin* transcript levels.

**Figure 4:**
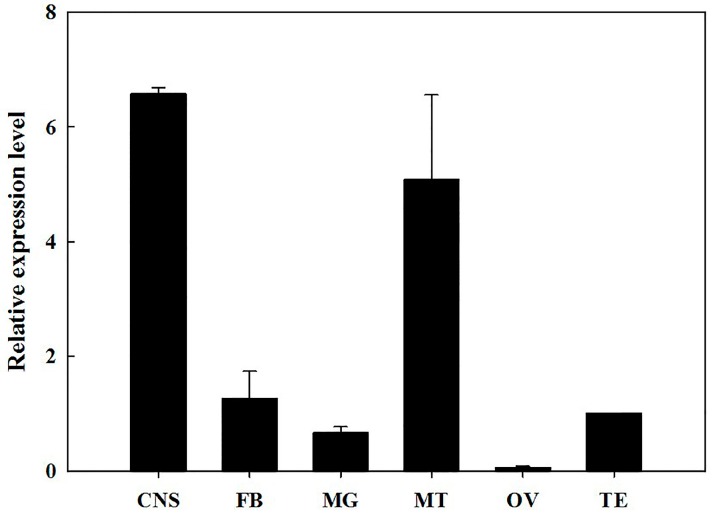
Relative expression levels of *BdOctβR1* in various tissues of adults. The data shown are mean ± S.E. (*n* = 3). Normalized relative to *α-tubulin* transcript levels. CNS: central nervous system; FB: fat body; MG: midgut; MT: Malpighian tubules; OV: ovary; TE: Testis.

The RT-qPCR results showed that *BdOctβR1* was expressed in all tested developmental stages, particularly in the larval and adult stages (Figure 3). The highest expression was seen in three-day-old adults and the lowest in eggs; the difference was 37.7 fold. During adulthood, the expression of *BdOctβR1* increased from day 1 to day 3, and then declined gradually from day 3 to 7. Over the different tissues of the adults (Figure 4), the highest expression levels of *BdOctβR1* were recorded in the central nervous system (CNS) and Malpighian tubules (MT), and the receptor was nearby absent in the ovaries.

### 2.3. Functional Expression and Concentration Responses of BdOctβR1 to Typical Ligands

**Figure 5:**
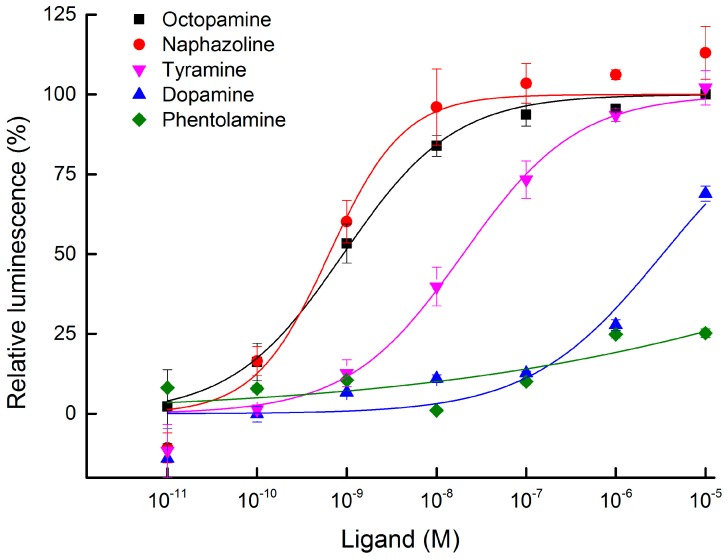
Agonist concentration-response curves of *BdOctβR1* transiently expressed in HEK-293 cells to five tested ligands (octopamine, naphazoline, tyramine, dopamine, and phentolamine). Each spot represents the mean relative luminescence ± S.E. from three independent tests and three biological replications per experiment. Each value was normalized to the cAMP luminescence obtained with octopamine at 1 × 10^−5^ M (=100%).

Transient expression of *BdOctβR1* was successfully carried out in HEK-293 cells transfected with the expression plasmid pcDNA3.1-BdOctβR1. In this system, ligand-mediated GPCR activation initiates cAMP accumulation that is measured by an increased luminescence of Glosensor. In our assay, we tested the concentration effects of five ligands: octopamine, tyramine, dopamine, phentolamine, and naphazoline. Octopamine and tyramine could significantly induce the increase of cAMP production in BdOctβR1-expressioning cells, but not in empty pcDNA3.1 vector-transfected cells (Supplementary Material, Figure S1). The results showed that octopamine, naphazoline, and tyramine activated the BdOctβR1 in a concentration-dependent manner with a respective median effective concentration (EC_50_) of 9.11 × 10^−10^, 6.35 × 10^−10^, and 1.97 × 10^−8^ M (Figure 5; Table S1). Dopamine weakly activated this receptor (EC_50_ = 3.32 × 10^−6^ M) compared with the other ligands. Phentolamine, an adrenoceptor antagonist, showed only very weak agonistic effects: about 20% at a very high concentration of 1.00 × 10^−5^ M.

### 2.4. Antagonist Assay of BdOctβR1

**Figure 6:**
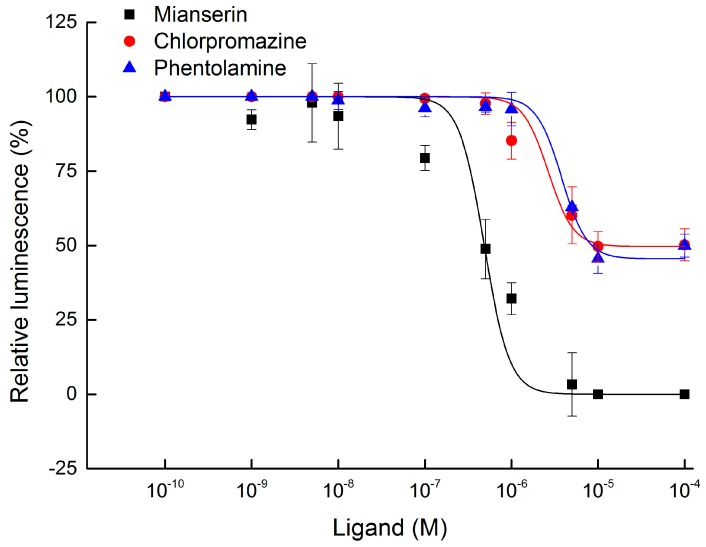
Antagonist concentration-response curves of BdOctβR1. Inhibition of the octopamine-dependent cAMP luminescence was examined on BdOctβR1-expressing HEK-293 cells with mianserin, phentolamine, and chlorpromazine. Each value was normalized to the cAMP luminescence obtained with the positive control, octopamine at 1.00 × 10^−6^ M (=100%). Mean values ± S.E. from three independent tests and three biological replications per experiment.

For the pharmacological characterization of BdOctβR1, we examined the effects of various potential antagonists on the octopamine receptor of *B. dorsalis*, including phentolamine, mianserin, and chlorpromazine. The antagonistic activity was quantified as described (in Materials and Methods). These data were used to construct concentration response curves. As shown in Figure 6, the most efficient antagonist of the octopamine-stimulated BdOctβR1 was mianserin with a median inhibitory concentration (IC_50_) of 4.84 × 10^−7^ M. The two other compounds, phentolamine and chlorpromazine, had a respective IC_50_ of 3.80 × 10^−6^ M and 2.66 × 10^−6^ M (Table S2). However, it should be remarked here that the most effect was only around 50% inhibition with the concentration tested (1.00 × 10^−5^ M). In the negative controls with empty pcDNA3.1 vector-transfected HEK-293 cells, all tested ligands at 1.00 × 10^−6^ M did not affect the cAMP luminescence.

### 2.5. Expression Profile of BdOctβR1 When Adult Flies Are under Stress

**Figure 7:**
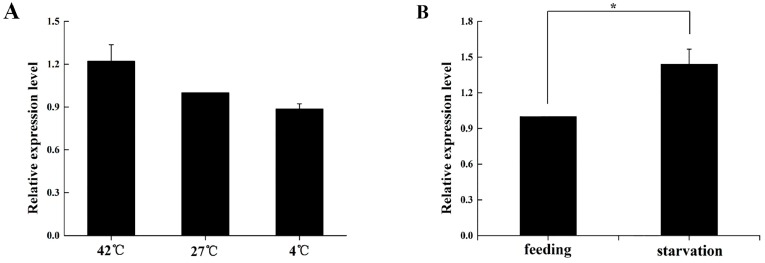
Expression profile of *BdOctβR1* when *B. dorsalis* adults were kept under thermal (**A**) or starvation stress (**B**). The data are means ± S.E. of three independent experiments. Asterisk (*) above indicates a statistical difference determined by the independent samples *t*-test (* *p* < 0.05).

After thermo-stress of the *B. dorsalis* adults, *BdOctβR1* exhibited no significant differences in expression. Compared with the control (27 °C), the transcript level of *BdOctβR1* showed a slight increase of 20% at 42 °C and a slight decrease of 12% at 4 °C (Figure 7A); however, these effects were not significant. Interestingly, when adults were starved for 24 h, the relative expression of *BdOctβR1* showed a significant (*p* < 0.05) increase of 44% over the controls (Figure 7B).

## 3. Discussion

Our phylogenetic analysis indicated that BdOctβR1 is a member of the class of insect β-adrenergic-like receptors, which are structurally similar to the vertebrate β-adrenergic receptors [3,5]. When the receptors are activated by adrenaline and noradrenaline, they induce the increased production of cAMP for the signal transduction.

In the present study, we found that *BdOctβR1* was highly expressed in adults and larvae, and a similar expression pattern of *DmOctβR1* has been observed in *D. melanogaster* [15]. Based on these, we believe that our results suggest that octopamine might play an important role during the stages of larval and adult of *B. dorsalis*. Moreover, *BdOctβR1* exhibited a high expression in the CNS of the adult brain. Similarly, the orthologous receptors were found to be highly expressed in the CNS in many insects such as *D. melanogaster* [16], *C. suppressalis* [2], *S. gregaria* [10], and *A. mellifera* [17]. This may indicate that octopamine plays a key role in the nerve system as a neurotransmitter. Similarly, *BdOctβR1* expression was relatively high in the MT as compared with the CNS in three lepidopteran species, including *Trichoplusia ni*, *Pseudaletia unipuncta*, and *Pieris rapae* [18]. In the desert locust *S. gregaria*, the highest transcript level of *SgOctβR* was found in the flight muscles followed by the CNS, and it has been determined to be associated with flight ability [10]. These results suggest that octopamine may have additional roles in other insect tissues next to the CNS. Therefore, we speculate that *BdOctβR1* plays an important role in the MT in *B. dorsalis*, based on the temporal expression profiles. Therefore, further investigations focusing on the MT in *B. dorsalis* should provide more information to elucidate the functions of *BdOctβR1*.

The result of the agonist assays was very similar to OctβR1 in *D. melanogaster* [3]. Generally, the rank order for the potency of the tested ligands was the same (naphazoline > octopamine > tyramine > dopamine). Octopamine was more potent in *B. dorsalis* than was *D. melanogaster* (EC_50s_: 9.11 × 10^−10^ M vs. 5.56 × 10^−9^ M) [3]; in *A. mellifera*, the EC_50_ of octopamine for AmOctβR1 was 4.39 × 10^−8^ M [17]. This suggests that BdOctβ1R might have a better coupling with Gs than DmOctβ1R or AmOctβR1, or it is also possible that our bioluminescence detection system showed a higher sensitivity.

Additionally, we examined the effects of several antagonist candidates which have been proven to be effective antagonists for insect octopamine receptors [3,9,19]. Among these ligands, mianserin, phentolamine, and chlorpromazine were effective antagonists for OctαRs [20,21,22,23,24], OctβRs [2,3,9], and TyrRs [25], respectively. In our antagonist assays, mianserin was the only chemical with a significant antagonistic activity on BdOctβR1 of *B. dorsalis*. This is consistent with a previous study [3], in which mianserin blocked DmOctβR1 binding to octopamine in *Drosophila*. In addition, mianserin also had antagonistic activity on OctβR2 [3], OctβR3 [3], and TyrR [19] in *Drosophila*. Moreover, mianserin has been identified as a very efficient inhibitor of AmOctβRs at low concentrations (IC_50S_: 5 × 10^−9^–3 × 10^−9^ M) [17]. Therefore, this suggested that mianserin could be a valuable antagonist in future experiments on pharmacological or insect behavior effects. Phentolamine, which is a traditional antagonist of α-adrenergic octopamine receptors, showed moderate agonistic activity on DmOctβR1 rather than antagonistic activity. In a previous study, phentolamine also acted as a full agonist with little antagonistic effects on DmOctβR2 of *D. melanogaster* [3] and BmOctβR2 of *B. mori* [9]. However, phentolamine acted as a partial agonist and had a significant antagonistic effect on CsOctβR2 of *C. suppressalis* [2]. The fact that phentolamine structurally has an imidazoline ring which is similar to the agonist naphazoline might be the reason why it showed dual effects on different octopamine receptors [2]. However, as the mechanism of antagonism to specific receptors is complex, we believe that the effects of phentolamine in different insects might vary. Additionally, chlorpromazine had a weak antagonistic activity for BdOctβR1 in our assay as its inhibitory reached only 50% at a high concentration of 1 × 10^−5^ M. This is consistent with the results in *Drosophila* that chlorpromazine is ineffective on DmOctβR1 [3]; in contrast, chlorpromazine has been reported as a significant antagonist for the CsOctβR2 of *C. suppressalis* [2] and BmOctβR2 of *B. mori* [9].

To date, different studies on the agonist or antagonist profile of octopamine receptors have been performed. These results had attracted the attention of many scientists to elucidate the pathways of formamidines with regard to an insecticidal action [17,19]. Furthermore, two octopamine receptor agonists, amitraz and chlordimeform, have been tested as synergists of selected novel insecticides to increase their lethal action against fourth-instar larvae of *Aedes* *aegypti* [26]. Meanwhile, the β-adrenergic-like octopamine receptors can raise the intracellular cAMP levels more sensitively than the α-adrenergic-like octopamine receptors in response to the classical insecticide chlordimeform [24]. These studies provide more evidence that the receptors could be considered as promising targets to develop novel insect control chemicals.

Octopamine has various roles in insects, such as inducing different effects on regulating larval locomotion and grooming [27], conditional courtship [28], and olfactory learning and memory [29] in *D. melanogaster*, feeding behavior in *Phormia regina* [30], and olfactory learning and memory in *A. mellifera* [31,32,33]. In this study, we found that food deprivation, compared with thermal stress, caused a higher expression of *BdOctβR1*. Similarly, various stress factors such as vibration and those that are optical, thermal and chemical could increase the octopamine level in *P. americana* [34,35]. As the specific receptor of octopamine, *BdOctβR1* showed a similar response to stress as food deprivation. We believe that there may exist an association between the increase of the *BdOctβR1* transcript level and inhibition of food uptake. Therefore, although the function of *BdOctβR1* has not been identified yet, we believe it could be involved in the regulation of feeding in *B. dorsalis*.

## 4. Materials and Methods

### 4.1. Insects

The laboratory colony of the oriental fruit fly *B. dorsalis* was established from pupae obtained from Haikou, China, in 2008. The insects were reared as previously described in a temperature controlled room at 27 ± 0.5 °C, 70% ± 5% relative humidity on a 14:10 h photoperiod (light:dark) in the Key Laboratory of Entomology and Pest Control Engineering, Southwest University, Chongqing, China [36]. Larvae were fed on an artificial diet consisting of sucrose, yeast, wheat, and corn flour. Adults were provided with artificial diets containing yeast power, honey, sucrose, vitamin C, and water [37]. The 3-day-old adults were used in this study.

### 4.2. Reagents

Tyramine hydrochloride, (±)-octopamine hydrochloride, dopamine hydrochloride, naphazoline hydrochloride, forskolin, chlorpromazine hydrochloride, phentolamine hydrochloride, and mianserin hydrochloride were all purchased from Sigma-Aldrich (St. Louis, MO, USA). To culture HEK-293 cells, DMEM/F12 medium, fetal bovine serum (FBS), fungizone, penicillin, and streptomycin were purchased from Gibco Cell Culture at Life Technologies (Grand Island, NY, USA). TransIT-LT1 Transfection Reagent was obtained from Mirus Bio LLC (Madison, WI, USA).

### 4.3. Total RNA/DNA Extraction

RNA was extracted from different developmental stages (eggs, larvae, pupae, and adults on days 1, 3, 5 and 7) and specific adult tissues (central nerve system: CNS; fat body: FB; midgut: MG; Malpighian tubules: MT; female ovaries: OV; and male testis: TE) from treated and control adults using Trizol reagent (Invitrogen Life Technologies, Carlsbad, CA, USA) according to the manufacturer’s protocol. The single-strand cDNA was synthesized by the PrimeScriptTM first-strand synthesis system (Takara, Dalian, China). RNA/DNA was quantified with a Nanovue UV-Vis spectrophotometer (GE Healthcare, Fairfield, CT, USA), and the quality was ascertained by the absorbance ratio of optical density (OD_260/280_) and OD_260/230_.

### 4.4. Molecular Cloning

Specific primers (Table 1) for nested PCR corresponding to the predicted sequences of *BdOctβR1* in *B. dorsalis* (GenBank: XP_011212557) were designed to obtain the full-length cDNA sequence. PCR was carried out using the high fidelity DNA polymerase with the following procedure: initial denaturation at 98 °C for 2 min, followed by 35 cycles of 98 °C for 15 s, 58 °C for 15 s, and 72 °C for 2 min, and final extension at 72 °C for 10 min. PCR products were separated by electrophoresis on an agarose gel (1.0%). The purified amplicons were cloned into the pGEM-T Easy Vector (Promega, Madison, WI, USA) and transformed into Trans5α chemically competent cell (TransGen Biotech, Beijing, China). The transformants were selected with Luria-Bertani agar plates containing 0.1% ampicillin, and positive clones were sequenced (Invitrogen, Shanghai, China).

### 4.5. Bioinformatics Analysis

Similar sequences were obtained by a BlastP search against the nonredundant protein database on NCBI (http://www.ncbi.nlm.nih.gov). Multiple sequence alignments were performed with DNAMAN v. 6.03 (Lynnon Biosoft, Vaudreuil, QC, Canada). Transmembrane helices were predicted using the TMHMM server (http://www.cbs.dtu.dk). The amino acids and locate motifs were predicted on the position identifiers proposed by Ballesteros and Weinstein [38]. A phylogenetic tree was constructed using ClustalW2 (http://www.ebi.ac.uk) and the neighbor-joining method with 1000 bootstrap tests. The resulting tree was displayed graphically using MEGA 5.0 [39]. The FMRFamide receptor in *D. melanogaster* served as an out-group.

### 4.6. Quantitative Reverse Transcription-PCR (RT-qPCR)

RT-qPCR was performed on a Stratagene Mx3000P system (Stratagene, La Jolla, CA, USA) using iQ-SYBR Green Supermix (Bio-Rad, Hercules, CA, USA). The 20-μL reaction system contained 10 μL of SYBR Green Supermix, 7 μL of nuclease free water, 10 μM of each primer, and 500 ng of cDNA. The PCR conditions were 95 °C for 2 min, and 40 cycles of 95 °C for 15 s and 60 °C for 30 s. A melting curve analysis (60–95 °C) was carried out at the end of procedure to ensure the specificity of each pair of primers. In addition, *α-tubulin* (GU269902) was used as an internal reference based on previous evaluations [37]. For the samples collected from each developmental stage or tissue, we performed three biological replicates, and the data were analyzed with the 2^−∆∆*C*t^ method [40].

### 4.7. Heterologous Expression and Functional Assay

Constructs for transient expression in HEK-293 cells were made by conducting a NotI digestion for both pGEM-T-BdOctβR1 and pcDNA3.1(+) (gift from Dr. Yoonseong Park in Kansas State University, Manhattan, KS, USA). The NotI digested BdOctβR1 and pcDNA3.1(+), and they were then ligated by T4 DNA ligase (Promega) yielding pcDNA3.1-BdOctβR1. The correct insertion was confirmed by DNA sequencing. For the cAMP response assays, we used the non-lytic GloSensor cyclic AMP assay kit (Promega) for monitoring intracellular cAMP in live cells. At the same time, the empty pcDNA3.1 vector was transfected in HEK-293 cells as a negative control. The methods for transient expression of *BdOctβR1* in HEK-293 cells and procedures for the assays were followed as previously described [41,42,43,44]. Thirty hours after the transfection, the cells were collected and preincubated with 1% GloSensor substrate in HEK-293 cells (Invitrogen) before the test. Ligands being tested were plated in each well of the 96-well plate. As described previously, the cells were injected into a well, and the changes in luminescence were monitored [45].

A 10-fold serial dilution of the ligands octopamine, tyramine, dopamine, naphazoline, and phentolamine was applied to the cells. Forskolin (1.00 × 10^−5^ M) was used as a positive control. The elevated luminescence caused by cAMP accumulation was measured over a period of 15 min in 30-s intervals using an Orion microplate luminometer (Berthold Technologies, Bad Wildbad, Germany). The authentic ligand octopamine was selected as the model ligand for normalization of the luminescence. A concentration–response curve was generated for each receptor by logistic fitting in Origin 8.6 (OriginLab, Northampton, MA, USA) as previously reported [41,44].

The antagonist assay was examined with serial dilutions of antagonists in the transfected cells on a luminescence detection systems (Berthold Technologies). Basically, the antagonistic activity assay was conducted in 2 sequential treatments. In the 1st treatment, various concentrations of ligands (50 μL in volume) were plated in each well, including a solvent (DMEM, Dulbecco’s modified Eagle’s medium) as control. Following the injection of the cells into the well, a 30-min incubation with the tested ligands was conducted. Subsequently, the 2nd treatment was conducted. The cells were treated with 1.00 × 10^−6^ M octopamine, which was the minimum concentration inducing the maximal activity, and the luminescent response was measured. For the assays, the cells treated with DMEM in both the 1st and 2nd treatment were set as background. The cells were treated with DMEM in the 1st treatment, while 1.00 × 10^−6^ M octopamine in the 2nd treatment, without adding any antagonist, served as positive control. Relative luminescence of ligand was normalized to the largest positive control response in each plate (1.00 × 10^−6^ M octopamine) after background subtractions [43]. The values calculated in the treatment were used to construct the inhibitor concentration–response curves. Three compounds (phentolamine, mianserin, and chlorpromazine) were included in our antagonist assay. The assay was conducted with three biological replications, and each plate contained three technical replications of each ligand concentration.

### 4.8. Changes in BdOctβR1 Expression When Adult Flies Are under Stress

#### 4.8.1. Thermo-Tolerance Assay

For thermal treatments, 90 five-day-old flies were placed in testing chambers (MIR-154, Sanyo, Moriguchi, Osaka, Japan) at 42 °C for 1 h (high temperature) or 4 °C for 30 min (low temperature) to ensure test flies could recover. Flies kept at 27 °C served as controls. Artificial diet was supplied by inserting into the fly chambers a glass vial containing adult diet and a wet sponge for water supply. Subsequently, one live female and one live male were collected randomly, then frozen together in liquid nitrogen and stored at −80 °C until RNA extraction. RT-qPCR was performed as described above using the same primers that were used to study *BdOctβR1* expression. Three biological replicates were maintained for each temperature treatment.

#### 4.8.2. Food Deprivation

In the starvation experiments, we investigated the responsiveness after 24 h of food deprivation. In essence, 5-day-old flies were collected and fed after 12 h of starvation to ensure fly satiety, and the treatments were then deprived of food for a period of 24 h. In the control groups, the flies were provided with an artificial diet. After the starvation period of 24 h, the live flies were collected individually for sample preparation as stated above.

## 5. Conclusions

In summary, we characterized the molecular and pharmacological properties of *BdOctβR1*, which is a necessary step in elucidating the functions of octopamine receptors in *B. dorsalis*. Our study suggests that BdOctβR1 has potential as a target for the development of novel insecticides and is in relation to the regulation of feeding behavior, although the function of the receptor has not yet been fully determined. Therefore, further studies need to identify the functions of octopamine receptors in insect physiological processes, and this will provide more opportunities for discovering novel insect control agents.

## Figures and Tables

**Table 1:** Primers used for cloning; quantitative reverse transcription PCR (RT-qPCR) in *Bactrocera dorsalis*.

Application of Primers	Primer Names	Sequence (5′–3′)	Amplicon Length (bp)
ORF cloning	BdOctβR1-F1	AGCCGATGTATTTAGGTTTC	1377
	BdOctβR1-R1	TTTATTTTTAGTTCTGGTAGAGC	
	BdOctβR1-F2	ATGTATTTAGGTTTCATTGCG	1372
	BdOctβR1-R2	TTTTATTTTTAGTTCTGGTAGAGC	
RT-qPCR	BdOctβR1-qF	GCGCCATTTTTCCTATGGTA	150
	BdOctβR1-qR	TCCACGAAAATCACGATTGA	
	α-Tubulin-qF	CGCATTCATGGTTGATAACG	184
	α-Tubulin-qR	GGGCACCAAGTTAGTCTGGA	

## Supplementary Materials

Supplementary materials can be found at www.mdpi.com/1422-0067/17/10/1577/s1.
