# Supplementary material for: Characterization of a β-Adrenergic-Like Octopamine Receptor in the Oriental Fruit Fly, Bactrocera dorsalis (Hendel)
Source: Int J Mol Sci. 2016 Sep 22;17(10):1577. doi: 10.3390/ijms17101577 (PMC5085626; doi:10.3390/ijms17101577)
Supplement: Supplementary file 1 [file ijms-17-01577-s001.pdf]

Supplementary Materials: Characterization of a β-Adrenergic-like Octopamine Receptor in the Oriental Fruit Fly, *Bactrocera dorsalis* (Hendel)

Hui-Min Li, Hong-Bo Jiang, Shun-Hua Gui, Xiao-Qiang Liu, Hong Liu, Xue-Ping Lu, Guy Smagghe and Jin-Jun Wang

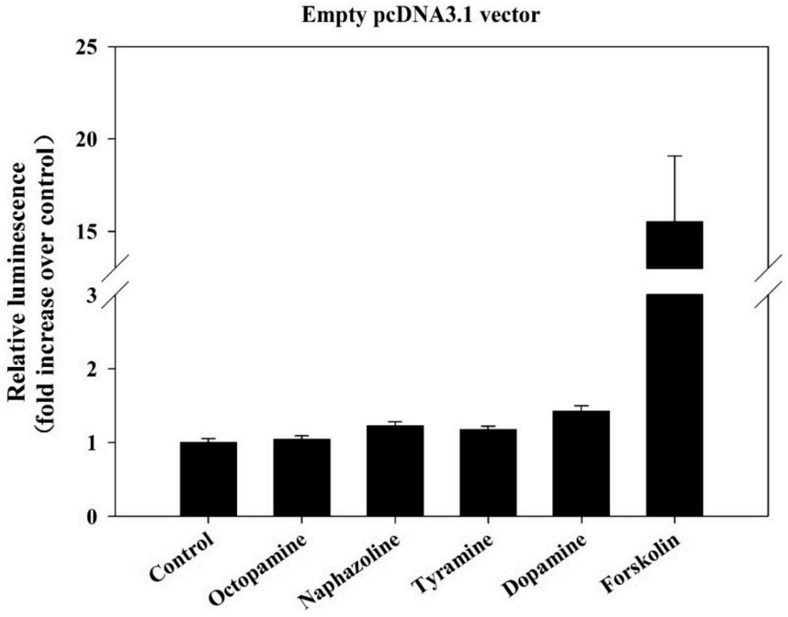

Figure S1. HEK-293 cells expressing empty pcDNA3.1 vector were challenged with indicated biogenic amines (1×10^{-6} M) or with forskolin (10×10^{-5} M) as a positive control. The negative control was Dulbecco's modified Eagle's medium (DMEM). Data represent means ± standard deviation (S.E.) from 6 experiments.

Table S1. Median effective concentration (EC_{50}) (mean ± S.E.) for ligands on BdOctβR1 in the agonist assay.

Ligands	EC_{50}(M)
Octopamine	$9.11 \times 10^{-10} \pm 0.99 \times 10^{-10}$
Naphazoline	$6.35 \times 10^{-10} \pm 2.02 \times 10^{-10}$
Tyramine	$1.97 \times 10^{-8} \pm 0.52 \times 10^{-8}$
Dopamine	$3.32 \times 10^{-6} \pm 1.43 \times 10^{-6}$
Phentolamine	$6.39 \times 10^{-3} \pm 2.30 \times 10^{-3}$

Table S2. Median inhibitory concentration (IC_{50}) (mean ± S.E.) for ligands on BdOctβR1 in the antagonist assay.

Ligands	IC_{50} (M)
Mianserin	$4.84 \times 10^{-7} \pm 1.12 \times 10^{-7}$
Chlorpromazine	$2.66 \times 10^{-6} \pm 0.48 \times 10^{-6}$
Phentolamine	$3.80 \times 10^{-6} \pm 0.35 \times 10^{-6}$
